# Unique patterns of cardiogenic and fibrotic gene expression in rat cardiac fibroblasts

**DOI:** 10.14202/vetworld.2020.1697-1708

**Published:** 2020-08-26

**Authors:** Kittipong Tachampa, Tuempong Wongtawan

**Affiliations:** 1 Department of Physiology, Faculty of Veterinary Science, Chulalongkorn University, Henri-Dunant Rd, Pathumwan, Bangkok, 10330, Thailand; 2 Akkhraratchakumari Veterinary College, Walailak University, Tha Sala, Nakhon Si Thammarat, 80160, Thailand; 3 Laboratory of Cellular Biomedicine, Faculty of Veterinary Science, Mahidol University, Salaya, Puttamonthon, Nakhon Pathom, 73170, Thailand

**Keywords:** cardiac fibroblasts, cardiomyocytes, gene expression, *in vitro* model, ischemia, reperfusion

## Abstract

**Background and Aim:**

Cardiac fibroblasts are important for both normal and pathological states of the heart, but the knowledge in cell physiology and genomics is still poorly understood. The aims of the present study were; first, to investigate the expression of cardiac and fibrotic genes in rat cardiac fibroblasts compared to cardiomyocytes and other fibroblasts (skin and muscle fibroblasts), second, to examine the in vitro effect of serum concentration on fibroblast gene expression. The findings can potentially be applied in ischemia/reperfusion models.

**Materials and Methods:**

Rat cardiac fibroblasts were collected and cultured in different conditions, and their gene expression (21 cardiogenic genes and 16 fibrotic genes) was compared with cardiomyocytes and other fibroblasts using comparative quantitative polymerase chain reaction. We also mimicked myocardial ischemia/reperfusion by depleting and then adding a serum into the culture in conventional culture (10% serum).

**Results:**

Cardiac fibroblasts expressed most of the cardiogenic genes, but their expression levels were significantly lower than in cardiomyocytes, while almost all fibrotic genes in the cardiac fibroblasts were significantly more highly expressed than in cardiomyocytes, except matrix metallopeptidase 9 (Mmp9) which also had greater expression in other fibroblasts. After mimicking cardiac ischemia and reperfusion in vitro by starving and then adding a serum into the cardiac fibroblast culture, the results revealed that Mmp9 expression was significantly increased (>30 times) after increasing but not reducing the serum in the culture. The expression of most cardiogenic and fibrotic genes in cardiac fibroblasts tended to decrease after increasing the serum in the culture. These changes were specific to cardiac fibroblasts but no other fibroblasts.

**Conclusion:**

Cardiac fibroblasts have a distinct pattern of gene expression from other fibroblasts and cardiomyocytes. They are also sensitive to high serum concentration but not affected by serum depletion, suggesting that the process of developing cardiac fibrosis might be stimulated by reperfusion or overcirculation rather than ischemia. The cell starvation followed the adding of serum may serve as a useful model to study cardiac fibrosis cause by the change of blood flow.

## Introduction

The heart consists not only of cardiomyocytes, but cardiac fibroblasts are also a major component of heart tissue. They take up over 60% of the total number of cells, or approximately 30% of the heart tissue, in adult rats and humans [1-4]. It is believed that cardiac fibroblasts have an important role in the development, structural integrity, normal functioning (such as cell signaling and electrophysiology), pathological states, and remodeling of the heart [5-8]. Fibroblasts, the mesenchymal origin cells that are widely distributed in most tissues, produce components of the extracellular matrix (ECM) such as collagen, and glycosaminoglycans and can be activated by pro-inflammatory cytokines and growth factors causing tissue fibrosis [9]. Fibrosis (also known as scar tissue) is the connective tissue deposition that occurs after tissue injury as part of normal healing, but if the process is excessive, it can cause organ abnormalities [5]. Cardiac fibrosis is the imbalance of ECM production and degradation, contributing to cardiac dysfunction and death, usually due to myocardial infarction or heart attack [7]. It is well-known that heart disease is the leading cause of death in humans and dogs. Therefore, studying the biology and physiology of fibroblasts is important for understanding the disease and discovering novel treatments for tissue fibrosis, including cardiac fibrosis and myocardial injury.

A recent report comparing the expression of mouse cardiac fibroblasts to heart tissues and tail fibroblasts suggested that mouse cardiac fibroblasts are unique, and express some cardiac markers such as *Gata4*, *Mef2c*, and *Tbx20* [10,11]. However, using heart tissue in the experiment had disadvantages. Namely, heart tissue contains too many cell types, including cardiomyocytes, endothelium, fibroblasts, and nerve cells. Therefore, the direct comparison of gene expression between cardiac fibroblasts and cardiomyocytes cannot be made using heart tissue. Moreover, the knowledge of property and function of cardiac fibroblasts is still limited. The rat is a valuable animal research model for investigating cardiovascular diseases. Many breeds and experimental procedures have been developed for disease studies, including myocardial infarction (MI), hypertension, cardiac hypertrophy, heart failure, and stroke [12]. The rat and primate cardiac fibroblasts are similar in biology and function and have been widely used to investigate both physiology and processes of cardiac diseases as a study model for human heart disease [5,6,8,13].

The aims of the present study were; first, to investigate the expression of cardiac and fibrotic genes in rat cardiac fibroblasts compared to cardiomyocytes and other fibroblasts (skin and muscle fibroblasts), second, to examine the *in vitro* effect of serum concentration on fibroblast gene expression. The findings can potentially be applied in ischemia/reperfusion models.

## Materials and Methods

### Ethical approval

This research project was approved by the Animal Care and Use Committee of Mahidol University (Approval Number: MUVS-2015-43). Rats were obtained from the National Laboratory Animal Centre, Mahidol University. In total, six Sprague Dawley rats (*Rattus norvegicus*) weighing between 250 and 300 g were used. The first group of three rats was used to collect cardiomyocytes and the remainings were used to obtain cardiac fibroblasts.

### Experimental plan

The expression of cardiac and fibrotic genes was examined through three different experimental studies. In the first study, a comparison of gene expression between cardiac fibroblasts and cardiomyocytes was performed, followed by a comparison of gene expression among fibroblasts from different sources: Skin, skeletal muscle, and heart. In the second study, the effects of serum concentration on gene expression in cardiac, skin, and muscle fibroblasts were analyzed and compared. In the last experiment, we investigated gene expression in fibroblasts in an experimentally-simulated myocardial ischemia/reperfusion using an *in vitro* culture system. The procedure involved initial starvation by providing no serum to the culture, then subsequently adding a serum to the culture.

### Single cardiac myocyte isolation

Ventricular myocytes were isolated using procedures modified from a previous study [14]. Rats were first anesthetized by pentobarbital sodium (Nembutal, CEVA, Belgium) at a dosage of 40 mg/kg, injected intraperitoneally. The thorax was surgically opened, then the heart was detached and immediately transferred to a dish containing 10 mL of perfusion buffer (133.5 mM NaCl, 4 mM KCl, 1.2 mM NaH_2_PO_4_, 10 mM HEPES, 1.2 mM MgSO_4_, 33.33 mM dextrose and 0.1% BSA). Thereafter, the heart was cannulated and perfused with perfusion buffer for 4 min (the flow rate was 3 mL/min) to remove the remaining blood clot within the chamber. Then, cardiomyocyte digestion buffer containing 0.05% collagenase type II (Merck, MO, USA) and 0.02% protease IV (Merck, Missouri, USA) was perfused for 10 min. The obtained cell suspension was transferred into a 15-mL tube before inactivating proteases by adding 2.5 mL of myocyte stopping buffer containing serum. To enrich the cell suspension with viable cardiomyocytes and to remove large, undigested chunks of tissue, dispersed cardiomyocytes were filtered through a nylon mesh (cell strainer) with a hole size of 200-300 μm (BD Bioscience, CA, USA). Samples were centrifuged at 5.41×g for 1.5 min, and then the supernatants were removed. Cell pellets were kept at −80°C for further RNA extraction.

### Fibroblast isolation and culture

The rats were euthanized by intraperitoneal injection of sodium pentobarbital (150 mg/kg). Fibroblasts were isolated from three tissue types: Skin (ear), muscle (hind limb), and heart (ventricle). Tissues of the same type from the three rats were mixed together. The tissues were cut, washed in phosphate-buffered saline (PBS), and minced in a 250 μL drop of 0.025% collagenase type I (Sigma-Aldrich, MO, USA) in a 100 cm tissue culture plate. The small pieces of tissue were transferred into a 50 mL sterile conical tube (Falcon^®^, Corning, NY, USA) containing 2.5 mL of 0.025% collagenase type I and incubated at 37°C for 20 min for the skin tissue, 35 min for the muscle tissue, and 50 min for the heart tissue. Then, 5 mL of 0.25% trypsin-EDTA (ThermoFisher Scientific, MA, USA) were added and incubated at 37°C for 10 min. Thereafter, 5 mL of PBS was added, and the tissues were vortexed briefly before being centrifuged at 1000×g for 5 min. Then, the sediment was resuspended with 5 mL of culture media containing Dulbecco’s Modified Eagle Medium (DMEM) with high glucose (Thermo Fisher Scientific, MA, USA), 10% fetal bovine serum (FBS) (Sigma-Aldrich, MO, USA), and antibiotic-antimycotic solution (Thermo Fisher Scientific, MA, USA). The cells were introduced into a 10-cm cell culture dish and incubated for 2 days at 37°C in a 5% CO_2_ incubator (Heal Force, Shanghai, China).

### Comparing the effect of serum concentration on gene expression in cardiac fibroblasts

Cardiac fibroblasts (the 3^rd^ passage) were cultured in three groups; a culture medium with varied serum concentration, 5%, 10%, or 20% FBS. Each group was cultured in a separated, six-well plate. Each plate contained the six replications of each treatment. They were cultured for a week before the analysis of gene expression.

### Serum starvation and adding serum

In this study, serum starvation was used as an *in vitro* model of ischemia, while adding serum after starvation was used as *in vitro* model of reperfusion.

Cardiac fibroblast culture was divided into four groups (four six-well plates). They were cultured in DMEM plus 10% FBS for 3 days. The first group was cultured with DMEM plus 10% FBS until day 8, while others were initially starved for 24 h by culturing in non-serum DMEM medium. After the starvation, one group of the cultures was collected for RNA collection, and the other two groups received serum with either 10% or 20% FBS and continued the culture for 4 days before RNA extraction.

### Comparative quantification of gene expression

RNA extraction was performed using Quick-RNA™ MiniPrep (Zymo Research, CA, USA). The RNA concentration was measured using a Nanodrop 2000 machine (Thermo Fisher Scientific, MA, USA). The extracted RNAs were converted to cDNAs by Superscript VILO cDNA synthesis kit (Thermo Fisher Scientific, MA, USA). These cDNAs were used as a template for quantitative polymerase chain reaction (qPCR).

Real-time qPCR was performed using KAPA SYBR Fast qPCR master mix (2x) universal (KAPA Biosystem, MA, USA) and Rotor-Gene Q PCR machine (Qiagen, Hilden, Germany). The PCR reaction contained 10 μL of the master mix, 0.4 μL of each primer, and 1 μg of cDNA and was filled with PCR-grade water to 20 μL. Comparative gene expressions were analyzed using Rotor-Gene Q software (Qiagen, Hilden, Germany). The PCR program was set similar to a previous publication [15]: Enzyme activation at 95°C for 3 min, denaturation at 95°C for 3 s, and Annealing/extension/data acquisitions at 60°C for 30 s, then performed for 35 cycles. Melt curve analysis was performed to confirm the specific product; the program for melt curve analysis was increasing temperature from 60°C to 99°C using speed rate 4 s/1°C. The geometric averaging of two housekeeping genes, glyceraldehyde 3-phosphate dehydrogenase (Gapdh), and actin beta (Actb) was used as a reference gene for normalization [16]. The PCR was done in triplicate.

The 21 cardiogenic genes used in this study were: Myoglobin (*Mb*), T-Box Transcription Factor 20 (*TBX20*) myosin light chain 2 (*Myl2*), myosin light chain 3 (*Myl3*), myosin light chain 7 (*Myl7*), natriuretic peptide A (*Nppa*), creatine kinase type M (*Ckm*), potassium voltage-gated channel subfamily Q member 1 (*Kcnq1*), troponin T2 - cardiac type (*Tnnt2*), phospholamban (*Pln*), actinin-alpha 2 (*Actn2*), ryanodine receptor 2 (*Ryr2*), NK2 homeobox 5 (*Nkx2-5*), GATA binding protein 4 (*Gata4*), adrenoceptor beta 1 (*Adrb1*), myosin heavy chain 7 (*Myh7*), transcription factor 21 (*Tcf21*), heart and neural crest derivatives expressed 2 (*Hand2*), solute carrier family 8 member A1 (*Slc8a1*), GATA binding protein 6 (*Gata6*), and Wilms tumor 1 (*Wt1*).

The 16 fibrotic genes were: Matrix metallopeptidase 9 (*Mmp9*), collagen type I alpha 2 chain (*Col1a2*) transforming growth factor-beta receptor 1 (*Tgfbr1*), vinculin (*Vcl*), connective tissue growth factor (*Ctgf*), tissue inhibitor of metallopeptidase 1 (*Timp1*), discoidin domain receptor tyrosine kinase 2 (*Ddr2*), vimentin (*Vim*), platelet-derived growth factor receptor-alpha (*Pdgfra*), actin-alpha 2 (*Acta2*), collagen type V alpha 2 chain (*Col5a2*), hepatocyte growth factor (*Hgf*), SMAD family member 2 (*Smad2*), Thy-1 cell surface antigen (*Thy1*), Paxillin (*Pxn*), and Periostin (*Postn*).

The primer sequences are listed in Table-1.

**Table-1:** List of polymerase chain reaction primers.

No.	Gene name	Sequence (5’->3’)	Template strand	Temp. (°C)	Product size	Group
1.	*ACTN2*	TGGAATGGATTCGACGCACA	Plus	60.04	238	Cardiogenic
		CCAGCAATGTCCGACACCAT	Minus	60.68		
2.	*MYH7*	CTCAGTCATGGCGGATCGAG	Plus	60.32	222	Cardiogenic
		TTCACAGTCACCGTCTTGCC	Minus	60.53		
3.	*MYL2*	CCAGAACAGAGACGGCTTCA	Plus	59.68	257	Cardiogenic
		GCATCTCCCGGACATAGTCG	Minus	60.04		
4.	*MYL3*	CAGCTGAGCCTCTCAGGAAG	Plus	59.82	203	Cardiogenic
		GACAGAAAGGGTACCACGGG	Minus	60.04		
5.	*MYL7*	GTTCTCTCCTGCTGAGGTGG	Plus	59.75	101	Cardiogenic
		CCCCGTGGGTGATGATGTAG	Minus	59.89		
6.	*HAND2*	AAGAGGAAGAAAGAGCTGAATGA	Plus	57.50	127	Cardiogenic
		CCCAGACTCTTCACTGCTTGA	Minus	59.65		
7.	*NKX2-5*	GGGCGGATAAGAAAGAGCTG	Plus	58.41	110	Cardiogenic
		CACGCGTGGCTTCCGT	Minus	60.4		
8.	*ADRB1*	AGACGTGCTATGTGTGACGG	Plus	60.11	294	Cardiogenic
		ACGTAGAAGGAGACGACGGA	Minus	60.04		
9.	*NPPA*	GCAAACATCAGATCGTGCCC	Plus	59.9	170	Cardiogenic
		GGTGGTCTAGCAGGTTCTTGAAA	Minus	60.5		
10.	*RYR2*	TGAAGTCACAGGATCCCAACG	Plus	60.00	151	Cardiogenic
		AGCCACCATTGGTCCAGTTT	Minus	59.81		
11.	*KCNQ1*	GCCTCACTCATCCAGACTGC	Plus	60.46	186	Cardiogenic
		AGGACTCAGCCCGTTATCCT	Minus	60.03		
12.	*PLN*	ACAGAAGCCAAGGCCTCCTAAA	Plus	61.63	213	Cardiogenic
		GAGGTTCTGGAGGTTCTGACG	Minus	60.07		
13.	*SLC8A1*	AACCTCAGTGCCAGACACAT	Plus	59.23	272	Cardiogenic
		CTCCTATTTCTGGCCTCCGC	Minus	60.25		
14.	*CKM*	AAGGGTGGAGACGATCTGGA	Plus	59.96	145	Cardiogenic
		TGTTGAGAGCTTCCACGGAC	Minus	59.97		
15.	*MB*	ACAGGAAGTCCTCATCAGTCTA	Plus	57.68	240	Cardiogenic
		TCCAGGTACTTGACCGGGAT	Minus	59.96		
16.	*Tnnt2*	AACGACAACCAGAAAGTCTCCA	Plus	59.83	102	Cardiogenic
		CACAGGCAAGGAACAGAGCTT	Minus	60.82		
17.	*Gata4*	TGGCCAGGACTGTCGCTTC	Plus	62.24	235	Cardiogenic
		CTCCAGAAACTGCAGGAGGG	Minus	60.04		
18.	*Tbx20*	TCTTCACAGCAGTCACAGCC	Plus	60.25	130	Cardiogenic
		AGAACAAGATCTCATTCCCTCTCG	Minus	59.90		
19.	*Tcf21*	TCAACCTGACTTGGCCCTTT	Plus	59.44	219	Cardiogenic
		GGGATAGGGAGAGGAGCGAT	Minus	59.96		
20.	*Gata6*	CAACGCATGCGGTCTCTACA	Plus	60.74	296	Cardiogenic
		CAGAGCTGTTACCGGAGCAA	Minus	60.04		
21.	*Wt1*	CGGAAGCACACTGGTGAGAA	Plus	60.25	219	Cardiogenic
		CCGACAGCTGAAGGGCTTTT	Minus	60.89		
22.	*Col5a2*	CTGGAGCTGTTGGGCCATTA	Plus	60.03	187	Fibrotic
		ATGCCTTGAAGACCTGGTGG	Minus	59.96		
23.	*Postn*	TGCAAAAAGACACACCTGCAA	Plus	59.45	178	Fibrotic
		CCGAAGTCAATGGGGCTCTT	Minus	60.04		
24.	*Ddr2*	GCCAGTTTGGGGAGGTTCAT	Plus	60.25	101	Fibrotic
		CACCAGGACAGGCTGGTTAG	Minus	60.04		
25.	*Pdgfra*	AGGCTTGGGGCTCACTTTTT	Plus	60.11	132	Fibrotic
		CTCGGCCCTGTGAGGAGA	Minus	60.36		
26.	*Thy1*	TCCTGCTTTCAGTCTTGCAG	Plus	58.11	127	Fibrotic
		TCATGCTGGATGGGCAAGTT	Minus	59.96		
27.	*Acta2*	GGAGATGGCGTGACTCACAA	Plus	60.04	152	Fibrotic
		CGCTCAGCAGTAGTCACGAA	Minus	60.11		
28.	*Vim*	TGCGGCTGCGAGAAAAATTG	Plus	60.39	111	Fibrotic
		GGTCAAGACGTGCCAGAGAA	Minus	59.97		
29.	*Pxn*	GGAGGAACACGTCTACAGCTTC	Plus	60.67	224	Fibrotic
		AAATGATTGCTCGTCCCTCCG	Minus	60.74		
30.	*Vcl*	CCGCGATGCCGGTGTTT	Plus	61.15	261	Fibrotic
		TGCAAGCATTCTCGACCTTAATA	Minus	58.23		
31.	*Ctgf*	CTAGCTGCCTACCGACTGG	Plus	59.26	260	Fibrotic
		GGCTTGGCAATTTTAGGCGT	Minus	59.75		
32.	*Mmp9*	TCTGCCTGCACCACTAAAGG	Plus	59.96	288	Fibrotic
		CAGGCTGTACCCTTGGTCTG	Minus	60.04		
33.	*Timp1*	ACGCTAGAGCAGATACCACG	Plus	59.34	242	Fibrotic
		AGCGTCGAATCCTTTGAGCA	Minus	60.04		
34.	*Hgf*	ACAGCTTTTTGCCTTCGAGC	Plus	59.69	279	Fibrotic
		TGTCGGGATATCTTTCCGGC	Minus	59.61		
35.	*Smad2*	GCCGCCCGAAGGGTAGAT	Plus	61.55	164	Fibrotic
		TTCTGTTCTCCACCACCTGC	Minus	59.89		
36.	*Tgfbr1*	GGGGCGAAGGCATTACAGT	Plus	60.08	257	Fibrotic
		TGCTTTTCTGTAGTTGGGAGT	Minus	57.14		
37.	*Col1a2*	CTCTGGTGATCCTGGCAAAC	Plus	58.54	235	Fibrotic
		TTCACCGGGAAGACCCCTTT	Minus	61.06		
38.	*Gapdh*	AGCTCATTTCCTGGTATGACAA	Plus	57.43	258	Reference
		GGTATTCGAGAGAAGGGAGGG	Minus	59.04		
39.	*Actb*	CCACCAGTTCGCCATGGAT	Plus	60.08	249	Reference
		CAGTTGGTGACAATGCCGTG	Minus	60.04		

*Mb*=Myoglobin, *Myl2*=Myosin light chain 2, *Myl3*=Myosin light chain 3, *Myl7*=Myosin light chain 7, *Nppa*=Natriuretic peptide A, *Ckm*=Creatine kinase type M, *Kcnq1*=Potassium voltage-gated channel subfamily Q member 1, *Tnnt2*=Troponin T2 - cardiac type, *Pln*=Phospholamban, *Actn2*=Actinin alpha 2, *Ryr2*=Ryanodine receptor 2, *Nkx2-5*=NK2 homeobox 5, *Gata4*=GATA binding protein 4, *Adrb1*=Adrenoceptor beta 1, *Tcf21*=Transcription factor 21, *Hand2*=Heart and neural crest derivatives expressed 2, *Slc8a1*=Solute carrier family 8 member A1, *Gata6*=GATA binding protein 6, *Wt1*=Wilms tumor 1

### Statistical analysis

Data are shown as mean±Standard Error Mean, and one-way ANOVA with the least significant difference adjustment for multiple comparisons was performed using SPSS software version 21.0 (IBM Corp., NY, USA).

## Results

### Cardiogenic and fibrotic gene expression in cardiac fibroblasts compared to cardiomyocytes

**Figure-1:**
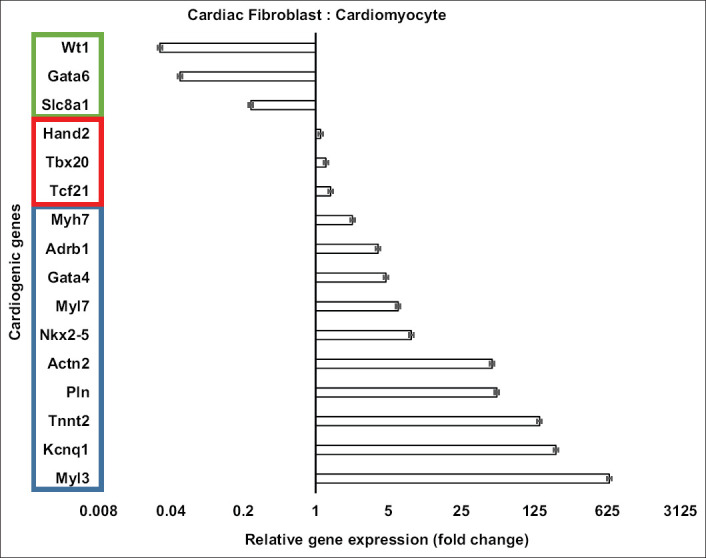
Expression of cardiogenic genes in cardiomyocytes compared to cardiac fibroblasts. Ten genes (in the blue rectangle) had greater expression in cardiomyocytes than in cardiac fibroblasts (p<0.01). Three expressed genes (in the red rectangle) were not different (p>0.05). Three genes (in the green rectangle) were more expressed in cardiac fibroblasts than in cardiomyocytes (p<0.01). Cell culture was done in six replications while quantitative polymerase chain reaction was done in three replications.

The expressions of cardiogenic genes are shown in Figure-1. From 21 cardiogenic genes, cardiomyocytes expressed all of the markers, whereas cardiac fibroblasts expressed only 16 genes. The expression of *Ryr2*, *Ckm*, *Nppa*, *Myl2*, and *Mb* was not detected in cardiac fibroblasts.

The expression of most cardiogenic genes (ten of 16 genes) was significantly higher (p<0.01) in cardiomyocytes than in cardiac fibroblasts. The expression of *Myl3*, *Kcnq1*, *Tnnt2*, *Pln*, and *Actn2* in cardiomyocytes was 50 to 660-fold higher than in cardiac fibroblasts. *Nkx2-5*, *Myl7*, *Gata4*, *Adrb1*, and *Myh7* in cardiomyocytes were expressed 2 to 10-fold higher than in cardiac fibroblasts. Expression of *Tcf21*, *Tbx20*, and *Hand2* was not different (p>0.05). Conversely, expression of *Slc8a1*, *Gata6*, and *Wt1* was significantly higher (p<0.01) in cardiac fibroblasts than in cardiomyocytes.

**Figure-2:**
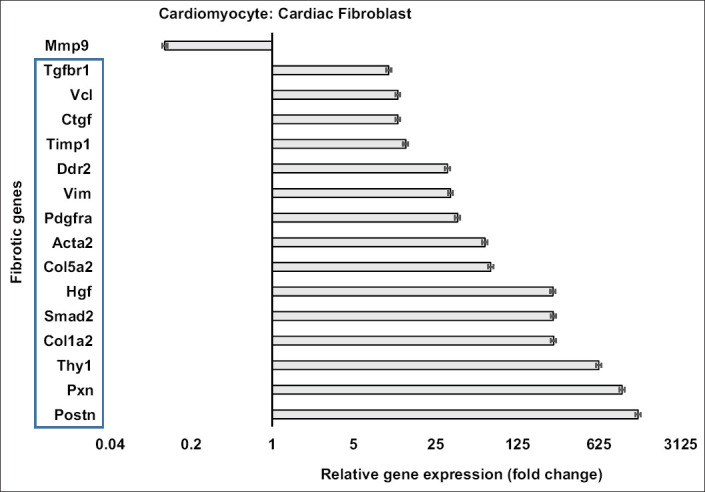
Fibrotic gene expression in cardiomyocytes compared to cardiac fibroblasts. Fifteen fibrotic genes (in the blue rectangle) had greater expression in cardiac fibroblasts than in cardiomyocytes (p<0.01). Matrix metallopeptidase 9 expression was significantly higher in cardiomyocytes than in cardiac fibroblasts (p<0.01). Cell culture was done in six replications while quantitative polymerase chain reaction was done in three replications.

The expressions of fibrotic genes are shown in Figure-2. Almost all of the fibrotic genes (15 of 16 genes) in cardiac fibroblasts were expressed significantly more than in cardiomyocytes (p<0.01), except *Mmp9*. The expression of *Postn*, *Pxn*, and *Thy1* was more than 500-fold higher in cardiac fibroblasts than in cardiomyocytes. The expression of *Col1a2*, *Smad2*, and *Hgf* in cardiac fibroblasts was approximately 200-fold higher than in cardiomyocytes. *Col5a2*, *Acta2*, *Pdgfra*, *Vim*, *Ddr2*, *Timp1*, *Ctgf*, *Vcl*, and *Tgfbr1* were expressed 10 to 75-fold higher in cardiac fibroblasts than in cardiomyocytes. On the other hand, *Mmp9* expression in cardiomyocytes was 8-fold higher than in cardiac fibroblasts (p<0.01).

### Comparing gene expression among fibroblasts from different sources

**Figure-3:**
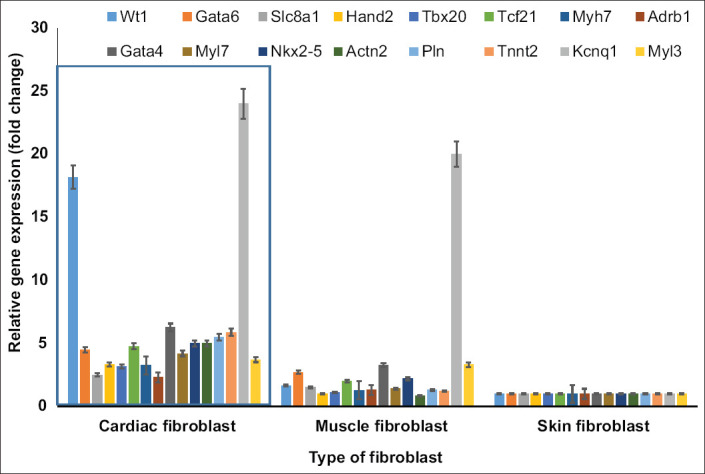
Comparative gene expressions of cardiogenic genes among cardiac, muscle, and skin fibroblasts. The gene expression of the skin fibroblasts was set to 1, and the gene expressions of muscle and cardiac fibroblasts were calculated relative to skin fibroblasts. All cardiogenic genes had higher levels of expression (p<0.01) in cardiac fibroblasts (in the rectangle) than in muscle and skin fibroblasts. The high expression of Wilms tumor 1 was very specific to cardiac fibroblast. Cell culture was done in six replications while quantitative polymerase chain reaction was done in three replications.

All cardiogenic genes were significantly more expressed (p<0.01) in cardiac fibroblasts, compared to muscle and skin fibroblasts (Figure-3). The very high expression of *Kcnq1* was specific to cardiac fibroblasts (24-fold) and muscle fibroblasts (20-fold) compared to skin fibroblasts. *Wt1* was highly expressed only in cardiac fibroblasts (18-fold).

**Figure-4:**
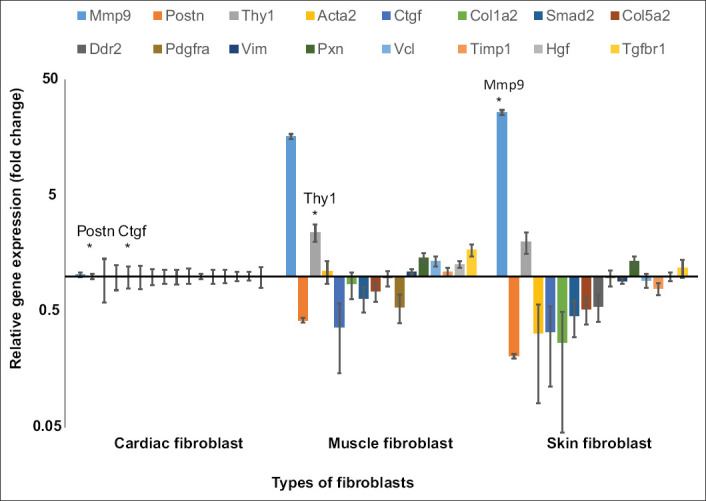
Relative gene expression of fibrotic genes compared among cardiac, muscle, and skin fibroblasts. The gene expressions of muscle and skin fibroblasts were calculated relative to cardiac fibroblasts (set to 1). *Represents the highest expression (p<0.01). The highest expression of matrix metallopeptidase 9 was found in skin fibroblasts. Periostin and connective tissue growth factor were highly expressed in cardiac fibroblasts. Thy-1 cell surface antigen was the most highly expressed in muscle fibroblasts. Cell culture was done in six replications while quantitative polymerase chain reaction was done in three replications.

Most fibrotic gene expressions were similar among the three fibroblasts, except for *Mmp9*, *Postn*, *Ctgf*, and *Thy1* (Figure-4). The *Mmp9* expression was highest in skin fibroblasts (25-fold) compared to muscle fibroblasts (16-fold) and cardiac fibroblasts (1-fold) (p<0.01). The highest expressions of *Postn* (5-fold) and *Ctgf* (4-fold) were found in cardiac fibroblasts, while the highest expression of *Thy1* (3-fold) was seen in muscle fibroblasts.

### High serum concentration depressed cardiogenic gene expression

**Figure-5:**
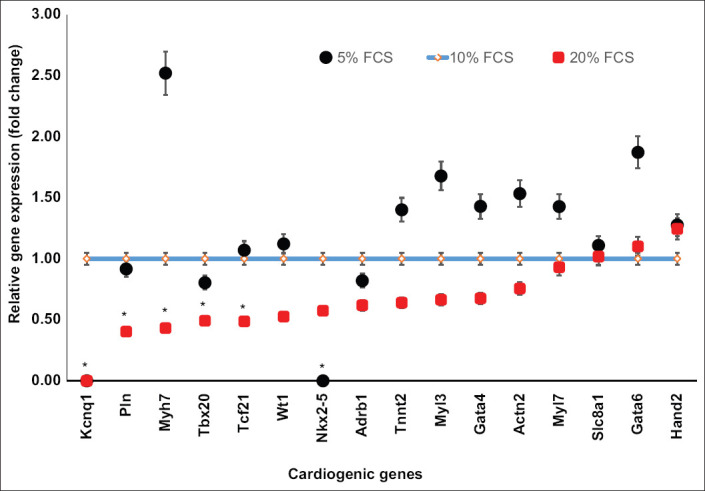
The effect of altered serum concentration in cultures of cardiac fibroblasts on cardiogenic gene expression. Gene expression of 10% fetal bovine serum culture (conventional culture) was set to 1, and the gene expression of culture in 5% and 20% serum was calculated relative to conventional culture. The result of reducing the serum to 5% in culture was variable, but adding the serum to 20%, tended to decrease gene expression of most cardiogenic genes. The expression of potassium voltage-gated channel subfamily Q member 1, phospholamban, myosin heavy chain 7, T-Box Transcription Factor 20, transcription factor 21, and NK2 homeobox 5 genes was significantly altered (p<0.05). Cell culture was done in six replications while quantitative polymerase chain reaction was done in three replications.

The effect of serum concentration on cardiogenic gene expression of cardiac fibroblasts is shown in Figure-5. After adding a serum to a level of 20%, the gene expressions of most cardiogenic genes (13 of 16 genes) tended to decrease, except for *Slc8a1*, *Gata6*, and *Hand2*. The result of reducing the serum to 5% was variable. When we considered only >2-fold change of gene expression with a statistical significance (p<0.05), *Kcnq1*, *Pln*, *Myh7*, *Tbx20*, *Tcf21*, and *Nkx2-5* showed a difference (6 of 16 genes). *Kcnq1* expression was undetectable after changing the serum concentration to both 5% and 20%, whereas *Nkx2-5* was unobtainable in 5% serum. *Myh7* expression was very high (2.52-fold) when cultured in 5% serum but its expression was 0.43-fold reduced when the serum media was switched to 20%. The expression of *Pln* (0.40-fold), *Tbx20* (0.49-fold), and *Tcf21* (0.49-fold) was lower in 20% serum.

### Effect of serum concentration on fibrotic gene expression in cultured cardiac fibroblasts

**Figure-6:**
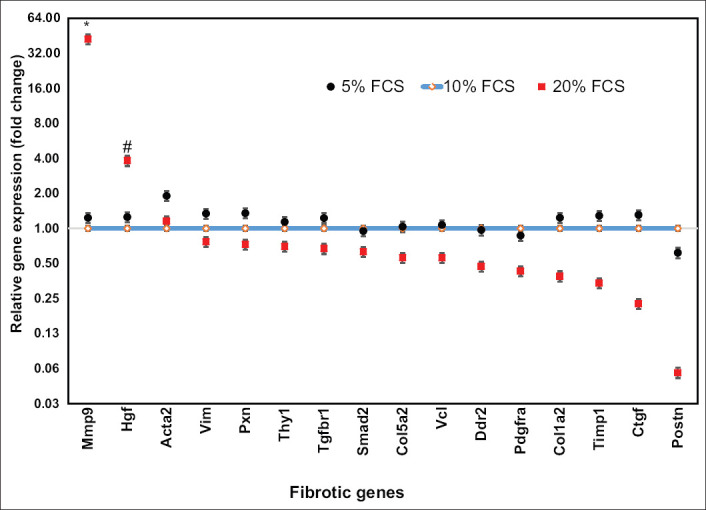
Effects of serum concentration on fibrotic gene expression. Gene expression of 10% fetal bovine serum culture (conventional culture) was set to 1 and the gene expression of culture in 5% and 20% serum was calculated relative to conventional culture. Increasing serum concentration to 20% resulted in a reduction of most fibrotic gene expression, except for matrix metallopeptidase 9 and hepatocyte growth factor, which was significantly increased (*p<0.01, # p<0.05). Cell culture was done in six replications, while quantitative polymerase chain reaction was done in three replications.

The expression of the fibrotic genes in cardiac fibroblasts is illustrated in Figure-6. The fibrotic gene expression of cardiac fibroblasts was not affected by reducing the serum in the system to 5%. Surprisingly, increasing serum concentration to 20% resulted in a reduction of most fibrotic genes’ expressions, except for *Mmp9* and *Hgf*, which were significantly increased 42.25-fold (p<0.01) and 2.82-fold (p<0.05), respectively.

### *In vitro* model of cardiac ischemia and reperfusion

**Figure-7:**
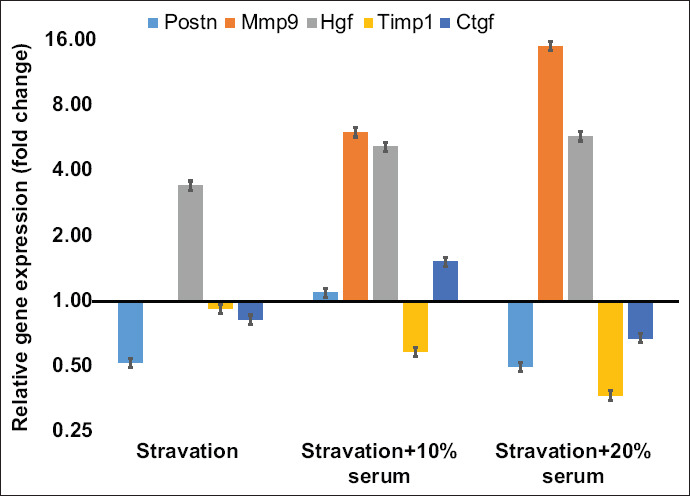
The effect of starvation and reintroduction of serum. Gene expression was calculated relative to a control culture (10% serum). Change of matrix metallopeptidase 9 and tissue inhibitor of metalloproteinase 1 expression in cardiac fibroblasts was statistically altered (p<0.05) by increased serum concentration, whereas hepatocyte growth factor expression was significantly altered (p<0.05) by both starvation and elevated serum concentration. Cell culture was done in six replications, while quantitative polymerase chain reaction was done in three replications.

It is well-known that an increase of *Mmp9* expression is related to heart disease, particularly myocardium ischemia [17,18] caused by coronary occlusion and resulting in cardiac cell starvation and injury. We mimicked blood blockage *in vitro* by removing serum in the culture medium for 24 h and investigated patterns of gene expression in five fibrotic genes (*Mmp9*, *Postn*, *Hgf*, *Timp1*, and *Ctgf*). The expression of genes in all three treatments (starvation and adding serum) was normalized by control culture (set to 1-fold). After the starvation, *Mmp9* expression was reduced to undetectable levels. Its expression was, however, significantly elevated (p<0.05) after the serum of either 10% (2.89-fold) or 20% (4.59-fold) sera was added into the environment (Figure-7). *Hgf* expression was 3.45-fold increased after the starvation, then substantially increased (p<0.05) after the addition of 10% (5.20-fold) or 20% (5.80-fold) sera to the system (Figure-7). *Timp1* expression was not affected after starvation, but its expression was significantly (2.72-fold) reduced (p<0.05) when serum of up to 20% was added into the medium. This result suggested that the changes of *Mmp9* and *Timp1* in cardiac fibroblasts were stimulated by increasing serum concentration, but not by starving, while *Hgf* expression could be induced by both starvation and elevated serum concentration.

### Comparative gene expression among fibroblasts from different tissues

**Figure-8:**
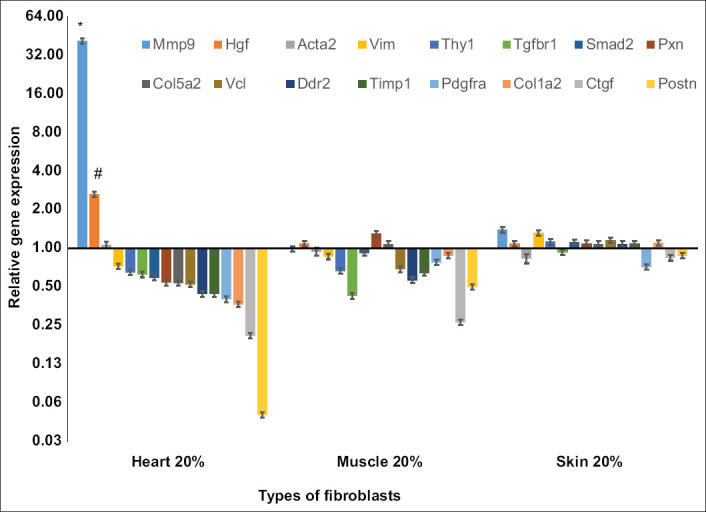
Fibrotic gene expression in 20% serum culture compared among three types of fibroblasts. The expression was calculated relative to 10% serum culture (control) and gene expression was relative to control culture (10% serum). Cardiac fibroblasts were more sensitive to increased serum concentration than skin and muscle fibroblasts. *The expression of most fibrotic genes from cardiac fibroblasts was reduced. Matrix metallopeptidase 9 and hepatocyte growth factor expression was significantly elevated (*p<0.01 and # p<0.05). Cell culture was done in six replications, while quantitative polymerase chain reaction was done in three replications.

We investigated whether this effect of high serum concentration was specific to cardiac fibroblasts, with no effect on other fibroblasts (e.g., skin and muscle fibroblasts). The gene expression of each type of fibroblast was calculated relative to conventional culture (10% serum). In 20% serum culture, each type of fibroblast behaved differently (Figure-8). Cardiac fibroblasts were found to be the most sensitive to the increased concentration of serum. Although the expressions of most of the fibrotic genes from cardiac fibroblasts were reduced, *Mmp9* and *Hgf* expressions were elevated approximately 40-fold (p<0.01) and 3-fold (p<0.05), respectively. Many fibrotic genes from muscle fibroblasts, particularly Tgfbr, *Ctgf*, and *Postn*, responded similarly to those from cardiac fibroblasts. In this study, we detected no effect of a high serum concentration on the gene expression of skin fibroblasts.

## Discussion

The present study has revealed that rat cardiac fibroblasts express both cardiogenic and fibrotic genes, but their expression patterns were different from cardiomyocytes and fibroblasts localized in other tissues. The expressions of most cardiogenic genes in cardiac fibroblasts were lower than those in cardiomyocytes. Notably, cardiac fibroblasts were more sensitive to a high serum concentration, compared to other fibroblasts. This resulted in notable changes in cardiogenic and fibrotic gene expression.

The expressions of cardiogenic genes such as *Slc8a1*, *Gata6*, and *Wt1* in rat cardiac fibroblasts were significantly higher than in cardiomyocytes. This finding is consistent with a previous study [10], where higher expressions of *Gata6* and *Wt1* were observed in mouse cardiac fibroblasts, compared to the heart tissue.

Considering a comparison of the fibrotic gene expression among the three types of fibroblasts, the present study has demonstrated that the *Mmp9* expression in cardiac fibroblasts was much lower than that in other fibroblasts and cardiomyocytes. This low level of *Mmp9* expression was previously reported in rats and humans [19,20]. Surprisingly, increasing serum concentration induced an extremely high level of expression of *Mmp9* in rat cardiac fibroblasts. It is well known that *Mmp9* plays a key role in the development of cardiovascular diseases as well as cardiac remodeling by altering ECM, fibroblast migration, collagen and cytokine production, and stimulating cell differentiation [18,21]. Increasing *Mmp9* expression in cardiac fibroblasts is a clinically detrimental sign as it is associated with matrix changes caused by heart disease [22]. *Mmp9* is also used as a marker to assess the severity of cardiovascular diseases [18,21] – an increased level indicates a poor prognosis. In myocardial ischemia, the increased levels of *Mmp9* protein in the serum are related to secretion of white blood cells such as neutrophils and macrophages [23,24]. Although cardiac fibroblasts are not a primary source for *Mmp9* production [23], moderately increased expression of *Mmp9* can, however, be observed in experimentally-induced myocardial ischemia model studies using hypoxia, oxidative stress, and cytokines such as interleukin-1β and tumor necrosis factor-alpha [20,25-27]. Nonetheless, the gene/protein expression in cardiac fibroblasts during myocardial ischemia/reperfusion is currently poorly understood. The majority of studies use an *in vitro* culture to investigate the physiology and molecular functions of cardiac fibroblasts [28]. In the present study, we used serum starvation/reduction and then added more serum to simulate myocardial ischemia/reperfusion phenomenon. Our results suggested that an introduction of the serum, but not after starvation, stimulated the high expression of *Mmp9*. This finding suggests a potential function of myocardial reperfusion being a key to induce *Mmp9* expression in survived fibroblasts and thus causing a rapid disease progression. In another study, one of the Mmp family proteins, Mmp1, is also found to increase after 20% of serum was added into the medium of human cardiac fibroblasts [29].

*Timp1* is a natural inhibitor of *Mmp9* [30], and it plays a regulatory role in post-myocardial ischemia by accelerating myocardial remodeling [31]. Both upregulation of *Timp1* and downregulation of *Mmp9* in cardiac fibroblasts have been proved to preserve cardiac functions and inhibit fibrosis in cardiomyopathy [32,33]. In this study, *Timp1* expression was not affected by a low concentration or absence of serum in the culture, but increased serum concentration could reduce the expression of *Timp1*. In a previous study, serum starvation was shown to stimulate other genes in the Mmp and Timp families, including Mmp2 and Timp2 [34].

As for *Hgf*, the gene expression was elevated both after starvation and after increasing the serum concentration in the culture to 20%. This result is similar to the previous *in vivo* studies [35,36]. The serum *Hgf* in these studies was heightened following acute myocardial ischemia/reperfusion. *Hgf* acts as a cardioprotective factor. The administration of *Hgf* protein was shown to reduce infarct size and promoted cardiac performance in acute ischemia/reperfusion in rats and dogs [36,37]. In contrast, a blockade of endogenous *Hgf* increases infarct size and mortality [37]. These cardioprotective effects of *Hgf* are proposed to be related to its angiogenic, anti-apoptotic, and anti-TGFβ1 pathways [37,38]. However, in another model of myocardial ischemia where cardiac fibroblasts were cultured in a hypoxic condition, *Hgf* was decreased [26].

It has been shown that high expression of *Ctgf* and *Postn* genes is important for heart remodeling after myocardial ischemia [39-43]. The function of *Ctgf* involves many cellular processes underlying fibrosis, such as cell proliferation, adhesion, migration, and the synthesis of ECM [41], while *Postn* primarily relates to cell adhesion and interactions with ECM [43]. The results from the present study illustrated that the levels of *Ctgf* and Potn were diminished after the serum withdrawal. Their expressions improved when the concentration of the serum was shifted to 10%, and declined after exposing to 20% serum. This phenomenon highlights the fact that ischemia can be responsible for a reduction of *Ctgf* and *Postn* expression in cardiac fibroblasts, but proper reperfusion can restore the expression of *Ctgf* and *Postn*.

While the effect of starvation on cultured cardiac fibroblasts has been commonly used to simulate myocardial ischemia [41,44-46], there has been only a limited number of studies simulating reperfusion injuries through an increased serum concentration in culture [29]. The strategy of using starvation and then introducing the serum can be useful to mimic myocardial ischemia and reperfusion injuries; however, actual cases of myocardial ischemia do not involve only nutrients, hormones, and proteins in serum but also a depleted oxygen condition. In most serum starvation studies, including the present one, oxygen persists in the system. This makes the model an imperfect simulation of myocardial ischemia/reperfusion *in vitro*. The culture of cardiac fibroblasts with hypoxia conditions has also been used as an alternative model for myocardial ischemia, and it produced the opposite result to the serum starvation technique. For instance, *Mmp9* expression was elevated, whereas *Hgf* expression was decreased [26].

## Conclusion

We propose that rat cardiac fibroblasts differ from other fibroblasts, in terms of their gene expression and sensitivity to serum concentration changes. An increased serum concentration, but not a reduced one, can substantially stimulate the expression of *Mmp9* and *Hgf*, while inhibiting *Timp1*, *Ctgf*, and *Postn*, suggesting that cardiac fibrosis may be stimulated by overwhelming of blood reperfusion rather than ischemia. The strategy of applying the starvation technique followed by a re-introduction of the serum could be methodologically useful in an *in vitro* study of the changes in blood flow to cardiac fibroblasts, which commonly occurs in myocardial ischemia/reperfusion cases.

Future studies should focus on the development of strategies to manipulate cardiac fibroblast to function properly. Specifically, seeking for new cardioprotective drugs that control fibroblast gene expression or optimal blood flow during reperfusion injuries.

## Authors’ Contributions

TW performed the experiment in cell culture and PCR while KT collected cardiomyocytes. KT and TW wrote manuscript and analyzed the data. Both authors read and approved the final manuscript.

## Competing Interests

The authors declare that they have no competing interests.

## Publisher’s Note

Veterinary World remains neutral with regard to jurisdictional claims in published institutional affiliation.
